# Curcumin Niosomes Prepared from Proniosomal Gels: In Vitro Skin Permeability, Kinetic and In Vivo Studies

**DOI:** 10.3390/polym13050791

**Published:** 2021-03-04

**Authors:** Tamer M. Shehata, Mahmoud M. Ibrahim, Heba S. Elsewedy

**Affiliations:** 1 Department of Pharmaceutical Sciences, College of Clinical Pharmacy, King Faisal University, Al Hofuf 31982, Saudi Arabia; helsewedy@kfu.edu.sa; 2 Department of Pharmaceutics, Oman College of Health Sciences, Pharmacy Program, Ministry of Health, Muscat 123, Oman; mahmoktar@yahoo.com

**Keywords:** curcumin, proniosomes, niosomes, emulgel, kinetics

## Abstract

Curcumin is a poorly water-soluble drug that is used for the treatment of inflammations, tumors, wound healing antioxidant and other diseases. In the current manuscript, it is successfully formulated into proniosome gels. The proniosomes are readily hydrated into niosomal formulations using warm water. Proniosomes were prepared using nonionic surfactants (tween 80, span 60) either solely or in combinations with cholesterol. The produced niosomal formulations were homogenous in size with vesicular sizes >343 and <1800 nm. The encapsulation efficiency percentage “EE%” of curcumin in niosomal formulations was different according to niosomal composition. It increased up to 99.74% in formulations of tween 80/Chol of 200 μmole/mL lipid concentration. Span 60/chol niosomes showed decreased curcumin EE%. Niosomal formulations showed increased SSTF and PC with enhancement ratios of more than 20-fold compared with curcumin suspension form. Kinetically, niosomes fitted to the Korsemeyer-Peppas model with non-Fickian transport according to their calculated n-values where curcumin suspension form showed Korsemeyer-Peppas kinetics with Fickian transport. Niosomal formulations deposited higher curcumin amounts in the skin compared with the suspension form. The best niosomal formulation (F9) was used for niosomal gel and emulgel fabrication. Finally, the anti-inflammatory activity of curcumin in various formulations was evaluated using a rat hind paw edema method and the % of swelling was 17.5% following 24 h in group treated with curcumin niosomal emulgel. In conclusion, this study suggests that the developed niosomal emulgel could significantly enhance the anti-inflammatory effect of curcumin and be an efficient carrier for the transdermal delivery of the drug.

## 1. Introduction

Diferuloylmethane or curcumin is obtained from turmeric, which is the powdered root of *Curcuma longa*. Curcumin possesses a wide spectrum of pharmacological effects, because of its inhibitory effects on some metabolic enzymes [1]. It inhibits cyclooxygenases (COXII) enzymes involved in inflammation via blocking the formation of reactive-oxygen species, which result in anti-inflammatory properties [2]. Additionally, curcumin disrupts cell signal transduction through the inhibition of protein kinase C and can play a role in the inhibition of tumor cell proliferation and inducing apoptosis [3,4]. Moreover, curcumin showed antioxidant, wound healing and antimicrobial effects [5]. Curcumin is poorly soluble in water and has poor gastro intestinal (GIT) absorption with reported poor bioavailability even at high drug dosage [6,7]. It is safe even at high dosage up to 12 g/day. It had been reported that no curcumin was detected in urine following oral administration where it showed high level excreted in the feces due to rapid metabolic elimination by reduction and conjugation [8]. Therefore, it is a challenge to achieve adequate curcumin plasma concentrations that could enhance its great desirable pharmacological benefits. Hence, a drug delivery system is required to achieve enhanced drug solubility and absorption, and decreased curcumin metabolism and elimination [9].

Niosomes are nano to several micrometer sized drug delivery systems that could enhance the solubility and bioavailability of poorly soluble drugs through their unique amphiphilic structures [10]. They are vesicular structures resembling liposomes in developing multilamellar and unilamellar vesicles except that they are with a nonionic surfactant backbone [11]. It is believed that lipophilic drugs are entrapped within the lipid bilayers where the water-soluble drugs are encapsulated in the water compartments of niosomes. This drug delivery system also protects drugs against chemical degradation and enhances drug physical stability. Furthermore, niosomal vesicles showed great stability against GIT enzymes and pH conditions compared to liposomes. The lymphatic pathway of absorption of niosomes had been proven by several studies that improved the oral bioavailability of several drugs [12,13]. As well, proniosomes are nonionic structured provesicles; however, they are in the form of gel states, nonaqueous solutions, or powdered forms. They transform quickly into niosomal vesicles upon hydration [14,15]. They offer more physically stable drug carriers where no aggregations, flocculation, or creaming could occur, hence they offer longer shelf life. They can be reconstituted immediately into niosomal forms at time of administration. Some of the proniosoms formulations such as gel states were used for transdermal and topical drug delivery as shown by Vora et al., Alsara et al., and others [14,16].

Emulgel is referred to a transdermal drug-delivery system formed when gel and emulsion are used in combination. Emulgels have the benefit of high drug release and help to increase skin permeability for drugs and consequently improve the drug efficacy [17].

The aim of the present manuscript was to incorporate the poorly soluble drug curcumin into niosomal formulations prepared from proniosomal gels via simple aqueous hydration procedures. Further, to formulate a drug delivery system composed of best niosomal formulation combined with gel or emulgel in order to maximize the anti-inflammatory effect of curcumin for transdermal applications.

## 2. Materials and Methods

### 2.1. Materials

Curcumin, Sorbitan monostearate (span 60), Polysorbate 80 (tween 80), and cholesterol (Chol; >99%) were purchased from Sigma Chemical Co., St. Louis, MO, USA. All other chemicals and solvents were of analytical reagent grade.

### 2.2. Methods

#### 2.2.1. Preparation of Curcumin Proniosomes

Proniosomes of curcumin were prepared according to the method reported by Mahmoud et al., with some modification [15]. In wide mouth vials, the accurate amounts of cholesterol and surfactants (tween 80, span 60, tween 80/span 60 1:1) were weighed. The total lipid concentration was adjusted to be 50, 150 and 200 µmol/mL in the finally prepared niosomes. The solvent propylene glycol was added as 400 mg followed by 160 µL phosphate buffered saline “PBS” (pH 6.8). Vials were tightly sealed and warmed in a water bath (55–60 °C) for 5 min (Shaker Water bath, GFL Laboratories, Burgwedel, Germany) until complete cholesterol dissolution. Then, 50 mg of curcumin was added and dissolved in the lipid mixture. Proniosomes were then left to cool at ambient room temperature and translucent yellow to creamy yellow gels were obtained for all formulations (Table 1).

#### 2.2.2. Preparation of Curcumin Niosomes

The obtained proniosomal gels were heated at 60 °C to convert into solutions. Hot proniosomal solutions were mixed with hot phosphate buffered saline of pH 6.8 at 60 °C and hand shaken for 5 min to form niosomal suspensions [18].

### 2.3. Microscopic Examination

Smears of curcumin proniosomes before reconstitution were examined using optical microscopy at 40× magnification and pictures were taken using a digital camera. Likewise, niosomal formulations were reconstituted using buffer pH 6.8 and centrifuged at 3000 rpm for 10 min to remove unencapsulated pellets of curcumin, then smears were examined and photographed under the same microscope at 40× magnification [19].

### 2.4. Analysis of the Particle Size

The niosomal or emulsion droplet size was determined using Malvern Zetasizer (Malvern Instruments, Malvern, UK) at 25 °C. Then, 0.1 mL of the niosomal preparations were diluted to 4 mL before measurements of particle size. It is important that no drug particles were suspended during measurements [20].

### 2.5. Entrapment Efficiency (EE) Determination

The prepared niosomes were centrifuged at 4 °C for 10 min at 3000 rpm in order to precipitate the un-encapsulated curcumin. The supernatant was centrifuged again at 12,000 rpm to pelletize the curcumin loaded niosomes. The supernatant was collected and niosomal pellets were washed twice with PBS and washes were also collected for further drug determinations. Niosomal pellets were dissolved using Triton-X100 and centrifuged at 3000 rpm (Centrifuge, Hettich 2205, Tuttlingen, Germany) to precipitate curcumin particles. The supernatant decanted and curcumin particles were dissolved in methanol and determined using a UV-visible spectrophotometer (Jenway, 6205, spectrophotometer, UK) at 450 nm [21]. All experiments were carried out in triplicate (*n* = 3). The percentage of drug entrapment efficiency (EE) was calculated as follows:

(1) $$\mathrm{Entrapment}\;\mathrm{efficiency}\;\%\;(\mathrm{EE}\%)\;=\;\frac{\mathrm{Determined}\;\mathrm{entrapped}\;\mathrm{Curcumin}\;\;}{\mathrm{Total}\;\mathrm{curcumin}}\;\times 100$$

### 2.6. Ex Vivo Study

#### 2.6.1. Preparation of Rat Skin

Wistar rats of about 250–300 g were obtained from the animal breeding center, Faculty of Science, King Faisal University, Saudi Arabia. The dorsal hair of the rats was carefully removed using a suitable depilatory cream then animals were killed using ketamine overdosage. Prior to the permeation study, the skin samples were hydrated in phosphate buffer (pH 6.8) at 4 °C overnight in a refrigerator.

#### 2.6.2. Curcumin Skin Permeation Study

**Figure 1:**
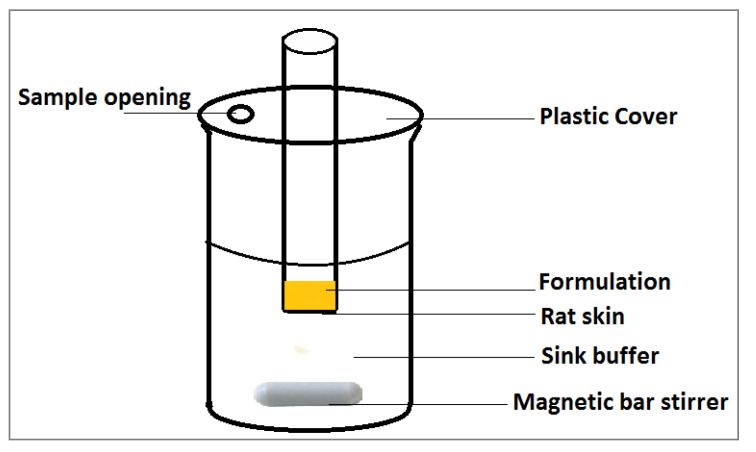
Diffusion cell for curcumin permeability study across rat skin.

In vitro skin permeation study of curcumin across male Wistar rat skin was performed using modified Franz diffusion cell constructed in our lab. The upper epidermis with the dermis portion of the skin was carefully separated and mounted on the diffusion cell “with surface area of 4.91 cm^2^” where the epidermis upside was facing the formulations to be permeated and the dermis portion was facing the acceptor buffer medium [22] (Figure 1). The sink medium was composed of 100 mL phosphate buffer (pH 5.5):methanol 1:1 ratio and was maintained at constant temperature (37 ± 0.5 °C) and the cell was covered to minimize methanol vaporization. The niosomal formulations or the curcumin suspension were applied to the donor area. Samples of 1 mL were taken over a period of 24 h at different time intervals of 0.5, 1, 2, 4, 6, 8, and 24 h. The aliquots were replaced by fresh medium to maintain the sink conditions [23]. The drug concentration was then determined using UV spectrophotometer at 450 nm. At the end of the permeation experiment the applied skin area was separated and washed twice with distilled water. The rat skin was homogenized into fine pieces and extracted using methanol by sonication. The curcumin extract was then filtered using 0.45-μm filter and was analyzed spectrophotometrically at 450 nm. Methanol filtrate of homogenized rat skin and sonicated with methanol was used as blank.

### 2.7. Kinetic Study

The permeation kinetics of curcumin was examined using kinetic models in which the drug amount (Q) is plotted against time (t) and Q_0_ is the initial drug concentration [24]. The kinetic models to be followed are as following:

Zero order equation: Q = Q_0_ − k_0_t (2)

where, a plot of (Q) against time (t) will be linear, if curcumin release obeys the zero order equation.

First order equation: Log Q= log Q_0_ − k_1_t/2.303 (3)

A linear plot of Log (Q) against time (t) will be obtained, if the curcumin release obeys the first order equation.

Higachi Kinetics equation: Q = K_H_t^0.5^ (4)

A linear plot of (Q) against the square root of time (t^0.5^) will be obtained if the curcumin release obeys the Higauchi equation.

Korsmeyer-Peppas release model equation: ft = K t^n^ (5)

where the fraction of curcumin permeation is (ft) at time (t), (k) is the permeation rate constant and (*n*) is the permeation rate exponent.

A linear plot of Log (% cumulative amount) against Log (time) will be obtained if curcumin permeation obeys the Korsmeyer-Peppas model equation.

### 2.8. Preparation of Curcumin Gel and Emulgel

**Figure 2:**
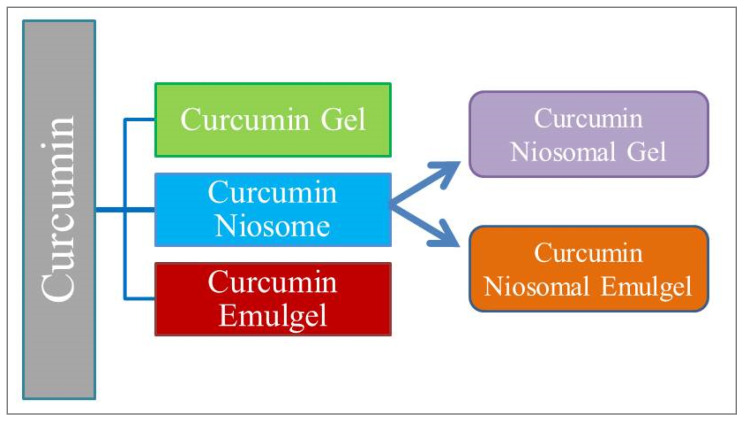
Schematic representation of prepared curcumin gel and emulgel.

Schematic representation of formulated curcumin niosomal gel and emulgel are shown in Figure 2 and compositions are represented in Table 2. The gels were developed by sprinkling sufficient amount of gelling gent consistently over a surface of water containing curcumin and mixing them for 10 min till a homogenous gel was attained. Regarding emulgel preparation, an oily phase (paraffin oil) was gradually added to an aqueous phase containing the surfactant (tween 80) and to which curcumin was dissolved. Afterward, the mixture was subjected to homogenization at 12,000 rpm for 5 min. Next, the essential amount of sodium carboxymethyl cellulose (NaCMC) was gradually added over curcumin-loaded emulsion and homogenized at 12,000 rpm for 10 min till a homogenous emulgel was formulated [25].

### 2.9. Preparation of Curcumin Niosomal Gel and Emulgel

In regard to niosomal gel or emulgel formulations, the amounts of all formulations are listed in Table 2. An equivalent amount of curcumin solution was substituted with curcumin loaded niosome preparation (F9) and the formulations were developed as previously mentioned.

### 2.10. Animals

Male Wistar rats with an average weight (220–250 g) were obtained from animal breeding center, college of science, King Faisal University and treated in accordance with “Ethical Conduct for Use of Animals in Research”. All animal experiments were performed according to the protocols approved by Animal Care Committee of King Faisal University (KFU-REC/2021-2-6). The animals were housed in a light and dark cycle in a controlled environment and an ambient temperature with free access to food and water.

### 2.11. Anti-Inflammatory Testing of the Prepared Formulae

The curcumin gel, emulgel and niosomal formulations of F9, that showed the highest in vitro skin permeation release, were tested for their anti-inflammatory activity using Carrageenan induced rat hand paw edema method. The rats were divided into six groups with five rats in each group as follow:

Group I: control group (induced with inflammation and receiving no treatment)

Group II: Placebo group (induced with inflammation and treated with niosomal emulgel free from the drug)

Group III: treated with curcumin gel formulation.

Group IV: treated with curcumin emulgel formulation.

Group V: treated with curcumin niosomal gel formulation.

Group VI: treated with curcumin niosomal emulgel formulation.

All rats received 50 μL of 0.5 % *w*/*v* carrageenan in saline into the left hind paw subcutaneously by inserting the needle into the central part of the paw. Test formulations were compared to the control group and placebo treated group. The inflammatory response was detected by measuring the changes in paw volumes using a digital caliber (Electronic digital Caliber, AHK, Germany) at different time intervals 0, 1, 2, 4, 6, 8, 12, and 24 h. The increase in paw thickness was measured at time zero and at different time intervals following carrageenan administration. The percentage of paw thickness increase from time zero was calculated. The % of swelling was calculated as reported by Khedr et al. [26] using the following equation:

Swelling (%) = [(Vt − V)/V] × 100

where Vt is the volume of carrageenan treated paw after specified time interval and V is the volume of injected hind paw at time zero.

### 2.12. Statistical Analysis

A one-way analysis of variance (ANOVA) followed by the least significant difference (LSD) as a post-hoc test, was applied using SPSS statistics software, version 9 (IBM Corporation, Armonk, NY, USA). The difference was considered statistically significant if *p* < 0.05.

## 3. Results and Discussion

Various preparations, namely curcumin niosome, curcumin gel, curcumin emulgel, curcumin niosomal gel, and curcumin niosomal emulgel were formulated successfully to estimate their capability to deliver curcumin through the skin.

### 3.1. Microscopic Examination

**Figure 3:**
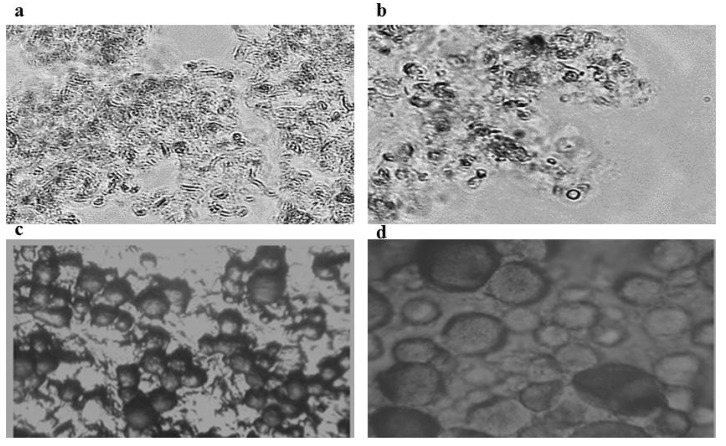
Photomicrographs of proniosomes (**a**,**b**) and reconstituted niosomes (**c**,**d**) of F7 and F9, respectively.

Proniosomes are anhydrous lamellar vesicles of a nonionic surfactant combined with cholesterol. The nonionic surfactant molecules direct their hydrophilic ends outward, while the hydrophobic ends are directed to the opposite direction to form the bilayer. Figure 3a,b photomicrographs show the proniosomes of F7 and F9, respectively. Small vesicular unilamellar and multilamellar structures are seen but the presence of proniosomes in the gel form makes it so close to each other and sometimes fused. On the other hand, following simple hydration by warm buffer, unilamellar vascular structures of F7 and F9 are well differentiated as presented in Figure 3c,d photomicrographs. Curcumin is a poorly soluble drug in both acidic and neutral conditions and the drug solubility increases in alkaline buffers, however, its stability is greatly affected via rapid hydrolytic degradation [27].

Proniosomes of curcumin were soft liquid crystalline gels with no precipitates as examined under light microscopes. This ensures that proniosome formulations have enhanced curcumin solubility in the form of semisolid gels. These formulations serve as precursors for niosome liquid colloids via simple hydration in warm distilled water or PBS. Moreover, proniosomes in the form of liquid crystalline gels are examined as successful transdermal patches by Vora et al. and Mahmoud et al. [14,15]. After reconstitution of proniosomes, some of the curcumin was pelletized and precipitated. The pelletized curcumin was removed by centrifugation at 3000 rpm for 10 min.

### 3.2. Analysis of the Particle Size

**Figure 4:**
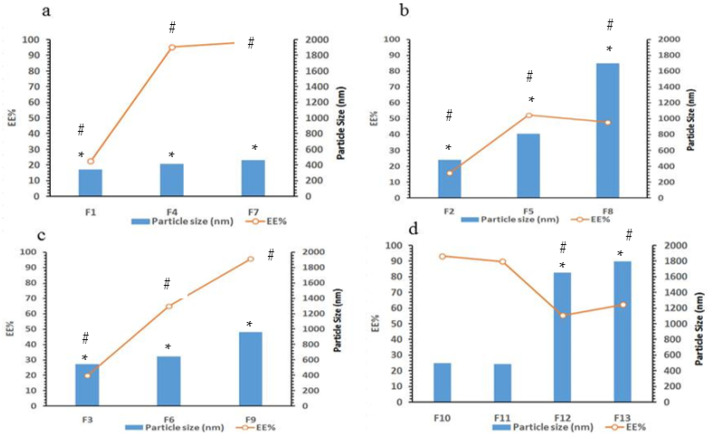
The EE % and particle size of curcumin niosomes in different formulations under investigations. (**a**) F1, F4, and F7, (**b**) F2, F5, and F8, (**c**) F3, F6, and F9 and (**d**) F10, F11, F12, and F13. (*) Significant change in particle size and (#) significant change in EE%.

The particle size of curcumin niosomes was estimated and results are listed in Table 3. It is interesting that niosomes were prepared by simple hydration of proniosomes by the addition of PBS of pH 7.4 at 60 °C. No further sonication or extrusion was required. The produced niosomes are of nano size with homogenous distribution as proven by polydispersity index (PDI) which is lower than 1 for the majority of preparations. As shown in Table 3 and Figure 4, the niosomal size is increased significantly (*p* < 0.05) as total lipids concentration increased from 50 to 200 umol/mL. Tween 80 produced niosomes of smaller sizes compared to span 60 niosomes, particle size ranges from 343 (F1) to 461 nm (F7) as tween 80 increased concentration from 25 to 100 μmol/mL, respectively. However, when span 60 concentration increased from 25 to 100 μmol/mL, a significant increase in vesicular size from 481 (F2) to 1701 nm (F8), respectively, was shown. A tween 80/span 60 1:1 mixtures of total concentrations of 25–100 µmol/mL were examined and results showed intermediate particle sizes produced ranging from 542 (F3) to 958 nm (F9), respectively. This indicates that tween 80 has negative impact on niosomes particle size while span 60 increases the particle size of niosomes.

Results in Table 3 revealed that cholesterol concentration could affect the vesicle size. Cholesterol is essential component in noisome formation especially when tween 80 is used as a surfactant. It increases the stability of the vesicles and participates essentially in bilayer packing. It is well known that without cholesterol some surfactants like tweens cannot form vesicles and formed micelles instead. Unlike tween 80, spans can form noisome vesicles without the need to add cholesterol [15,28]. However, cholesterol affects the homogeneity and vesicular stability to a great extent. Furthermore, increasing surfactant and decreasing cholesterol resulted in decreasing the vesicular size from 461 (F7) to 343 nm (F1) in case of tween 80 formulations, where the vesicular size increases from 1701 (F8) to 1800 nm (F13) in case of replacing tween 80 with span 60. This could be a result of changing the physical characteristics of the surfactant from liquid unsaturated with high HLB (tween 80, HLB = 15) to solid saturated with lower HLB (span 60, HLB = 4.7) [18].

### 3.3. Entrapment Efficiency Determination

The EE% of curcumin is clearly affected by surfactant type, total lipid concentration, and cholesterol content as presented in Table 3 and Figure 4. Tween 80 niosomes produced higher EE% when compared to niosomes made from span 60 surfactant. The longer unsaturated side chain oleate part of tween 80 could enhance the packing ability of the poorly water-soluble drug curcumin in noisome bilayers. Span 60 on the other hand is solid having the saturated stearic fatty acid side chain with a transition temperature of 53 °C. The complete curcumin solubilization was observed during proniosome preparations in both surfactants. However, following hydration, curcumin packing in niosomes containing span 60 was found lower than those containing tween 80. This could be due to the speculation that the transition of span 60 from liquid state at 60 °C to solid state at room temperature could lead to a decreased solubilization power for poorly water-soluble drugs and decreased packing effects compared to tween 80 surfactant. This also was noticed for the enhanced EE% of curcumin when combined surfactants (1:1, ratio) were used in preparing niosomes of curcumin. The powerful factor that increases the EE% of curcumin into niosomes was the total lipid concentration. Increasing total lipid content from 50 to 200 μmol/mL enhanced the EE% of curcumin for 3.3-, 4.9- and 6.3-fold using span 60, combined span 60/tween 80 1:1 ratio and tween 80, respectively, in preparing niosomes. A significant increase in the EE% was observed when cholesterol content decreased in case of span 60 (*p* < 0.05) where this factor had a nonsignificant effect when using tween 80 as surfactant (insignificant increase *p* > 0.05) (Table 3 and Figure 4). This might be due to the fact that both the drug and cholesterol are hydrophobic substances and packed in the bilayer structure, therefore, competition between cholesterol and curcumin for packing sites in the noisome bilayers could exist [18]. This revealed a plausible decrease in EE% when cholesterol content increased. This result was clear shown by span 60 niosomes where tween 80 results were insignificant statistically due to the high EE % which was nearly 100% for all formulations of 200 μmol/mL total lipids. Correlating the particle size parameter with that of EE% was clearly indicated in Figure 4. Calculations of the correlation coefficient resulted in positive correlation of 0.931 which means an increase in EE% of curcumin with increasing particle size in F1, F4, and F7 (Figure 4a). Similarly, the same result was observed with other formulations in Figure 4b,c with correlation coefficient of 0.625 and 0.924, respectively. For F10 and F11, decreasing cholesterol from 70 to 50 μmol, resulted in insignificant decrease in particle size of the produced niosomes (*p* > 0.05) and insignificant increase in EE % of curcumin. However, by replacing tween 80 by span 60 results are different. Decreasing cholesterol from 70 to 50 µmol in F12 and F13, respectively, resulted in a significant increase in niosomal particle size and curcumin EE% (*p* < 0.05) (Figure 4d).

### 3.4. Curcumin Skin Permeation Study

**Figure 5:**
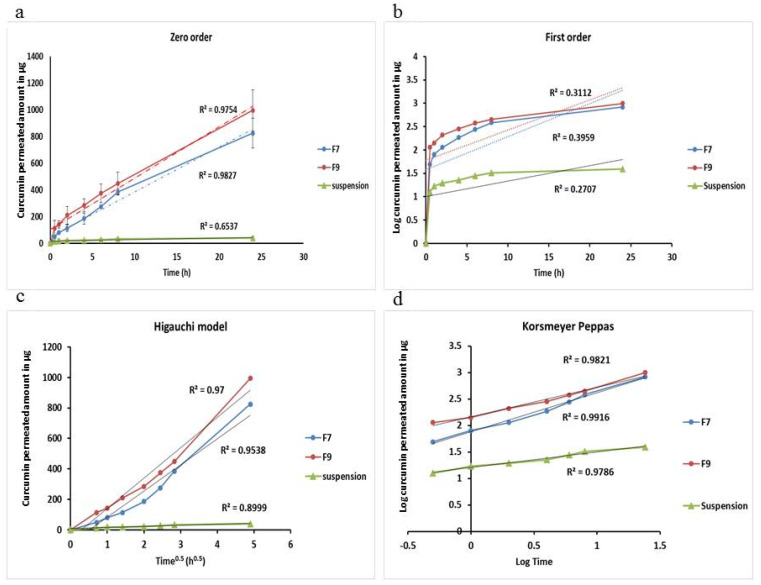
Amount of curcumin permeated from F7 and F9 versus curcumin suspension from rat skin and their kinetics according to zero order (**a**)- first order (**b**)- Higauchi model (**c**) and Korsmeyer-Peppas model (**d**).

The permeability of curcumin across rat skin from niosomal formulations is shown in Figure 5. F7 “not containing span 60” and F9 “containing span 60/tween 80 1:1 ratio” were selected for further study due to their high entrapment efficiencies for curcumin. It is clear that both F7 and F9 formulae are better than curcumin suspension. Both F7 and F9 are showing great permeability amounts compared to curcumin suspension and as calculated per SSTF and PC that were significantly different from curcumin suspension (*p* < 0.5). The enhancement ratio was 21.86 for F7 and 27.54 for F9 (Table 4). Interestingly, the SSTF and PC of F7 were found nonsignificantly different from those of F9 (*p* > 0.05).

### 3.5. Kinetic Study

Figure 5 and Table 5 show the different permeability kinetic mechanisms of zero order, first order, Higauchi “diffusion” model and Korsemayer-Peppas model for both F7 and F9 niosomal preparations compared with that of curcumin suspension. In order to determine the best permeability kinetics fitted by formulation under investigation, the most linear plot was proved according to the highest obtained R^2^ for each kinetic model. From Table 5 and Figure 5, it is obvious that the reconstituted niosomal formulations of F7 and F9 were found obeying Korsmeyer-Peppas model for curcumin release. Linear plots with highest R^2^ of 0.9916 and 0.9821 for F7 and F9, respectively, were obtained compared to other kinetic models. The permeability exponents (*n* values) for both formulations were found in the range of 0.45–0.89 [29]. When 0.45 < *n* < 0.89, where the mechanism of drug permeation is said to be non-Fickian transport that means a coupling of diffusion and erosion mechanisms or an anomalous permeation mechanism. In addition, curcumin suspension showed drug permeability obeying the Korsmeyer-Peppas model, but *n*-value is <0.45 which indicated Fickian transport mechanism [24].

**Figure 6:**
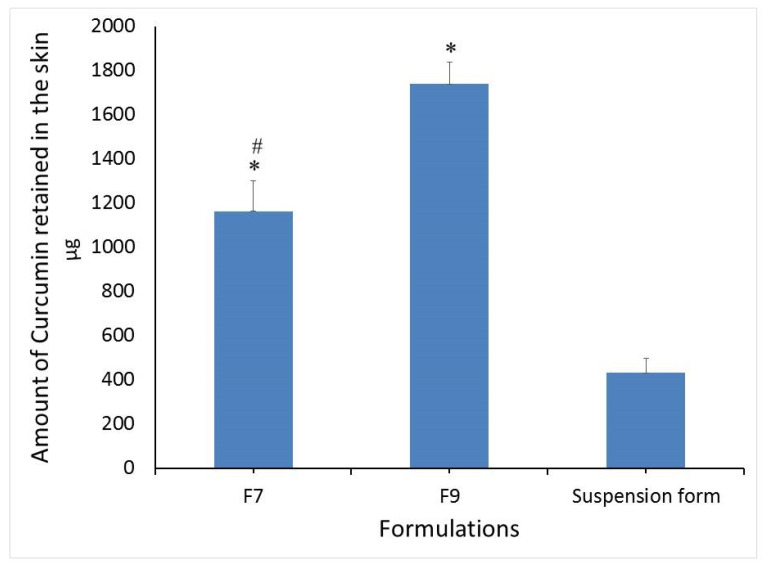
Amount of curcumin retained in rat skin following 24 h of permeability experiment. Values are expressed as mean ± (SD). * *p* < 0.05 compared to suspension form. # *p* < 0.05 compared to F9.

Figure 6 shows the amount of curcumin retained in rat skin after the completion of permeability study. It is clear that niosomal formulations showed significantly higher curcumin deposition into the skin by 2.69- and 4-fold for F7 and F9, respectively, compared with curcumin suspension form. This could be due to great permeability enhancing factor of niosomal components especially the nonionic surfactants. F9 also is showing significant high drug deposition compared with F7 (*p* < 0.05). This result might be due to the composition differences between the two formulations and due to the speculation that F9 contains span 60 in addition to tween 80. Span 60 is a more lipophilic component and could be retained at greater concentrations in the skin compared to tween 80.

### 3.6. Anti-Inflammatory Testing of the Prepared Formulae

**Figure 7:**
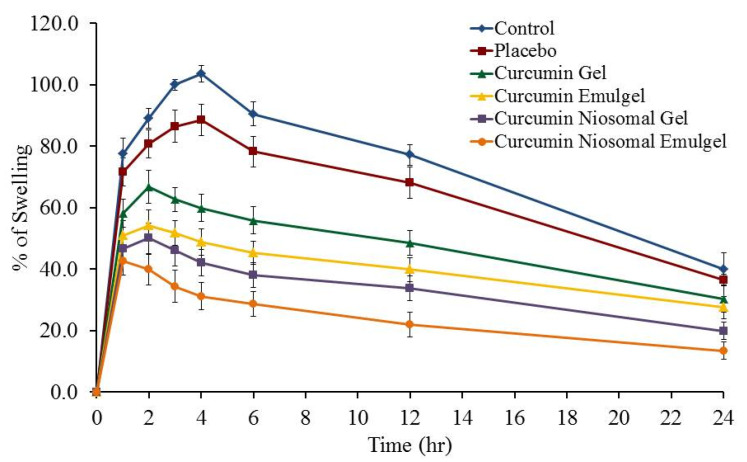
Effect of different curcumin formulations on carrageenan-induced hind paw edema in rats. Values are expressed as mean ± standard deviation (SD) *n* = 5.

The percentage of swelling following application of curcumin gel, curcumin emulgel, curcumin niosomal gel, and curcumin niosomal emulgel compared with control and placebo treated groups were measured as represented in Figure 7. The developed curcumin formulations exhibited a reduction in the volume of carrageenan-induced swelling of the hind paw edema, which suggests that the drug offered considerable effect. Injection of carrageenan resulted in a significant increase in the swelling of control and all treated groups to reach the highest inflammation after 4 h in control and placebo treated groups. Whereas, it reached the maximum inflammation in case of curcumin gel, curcumin emulgel, and curcumin niosomal gel treated groups after 2 h. On the other hand, maximum inflammation was achieved following 1 h of carrageenan injection in case of curcumin niosomal emulgel treated group. It is clearly noticed that, after 24 h, the effect of carrageenan declined and was insignificant for all groups under investigation. However, following 12 h the % of rat hind paw swelling decreased to 77.2 ± 3.3, 68.2 ± 3.3, 48.4 ± 5.1, 39.8 ± 4.0, 33.8 ± 4.0, and 22.0 ± 4.1% for control, placebo, curcumin gel, curcumin emulgel, curcumin niosomal gel, and curcumin niosomal emulgel treated groups, respectively. Curcumin niosomal emulgel group exhibited the highest prospective anti-inflammatory action, which is statistically significant (*p* < 0.05) compared to all other groups after 12 h. This could be ascribed to the better permeation of niosomal emulgel formulation owing to the presence of surfactant, oil and nanocarrier such as niosome [30]. Interestingly, the study comes up with clear evidence for the rapid onset of the niosomal emulgel formulation. Our results came in accordance with a recent study that proved the significant decline in percentage of inflammation in animals treated with curcumin loaded nanoemulgel compared with nontreated animals and placebo group [1,31].

## 4. Conclusions

The proniosomes method of preparation successfully enhanced curcumin EE % within the hydrated niosomal forms. Niosomal vesicles were small in size with homogenous distributions. Niosomal curcumin showed more than 20-fold increase in SSTF, PC and enhancement ratio when compared with non-niosomal curcumin suspensions. In addition, a significantly higher amount of curcumin was found deposited inside the rat dermis compared with that deposited from the suspension form. Niosomal formulations showed non-Fekian transport with high transport across rat skin compared to curcumin suspension. Moreover, this investigation revealed that the permeability and pharmacological effects of curcumin were synergistically increased upon developing combining niosomes of the drug with emulgel in one formulation.

## Figures and Tables

**Table 1:** The composition of proniosomal formulations.

Formulae	Tween 80 (µmol)	Span 60 (µmol)	Cholesterol (µmol)	PG (mg)	Curcumin (mg)	Proniosome Produced
**F1**	250	-	250	400	50	Translucent yellow gel
**F2**	-	250	250	400	50	Yellow creamy gel
**F3**	125	125	250	400	50	Yellow creamy gel
**F4**	750	-	750	400	50	Translucent yellow gel
**F5**	-	750	750	400	50	Yellow creamy gel
**F6**	375	375	750	400	50	Yellow creamy gel
**F7**	1000	-	1000	400	50	Translucent yellow gel
**F8**	-	1000	1000	400	50	Yellow creamy gel
**F9**	500	500	1000	400	50	Yellow creamy gel
**F10**	1300	-	700	400	50	Translucent yellow gel
**F11**	1500	-	500	400	50	Translucent yellow gel
**F12**	-	1300	700	400	50	Yellow creamy gel
**F13**	-	1500	500	400	50	Yellow creamy gel

**Table 2:** Composition of prepared curcumin gels and emulgels.

Materials	Curcumin Gel	Curcumin NIOSOMAL Gel	Curcumin Emulgel	Curcumin Niosomal Emulgel
**Curcumin(g)**	0.05	-	0.05	-
**NaCMC (g)**	0.8	0.8	0.8	0.8
**Liquid paraffin (mL)**	-	-	2.5	2.5
**Tween 80 (mL)**	-	-	0.25	0.25
**Niosomes loaded curcumin (mL) (best formulation)**	-	Q.s	-	Q.s
**Buffer pH 6.8 to (mL)**	20	20	20	20

Q.s: quantity sufficient.

**Table 3:** Results of vesicle size, PDI and EE %.

Formulae	Vesicle Size * (nm)	PDI	EE%
F1	343 ± 18	0.880 ± 0.150	22.50 ± 0.64
F2	481 ± 32	0.487 ± 0.128	15.75 ± 1.35
F3	542 ± 86	0.552 ± 0.465	19.68 ± 0.98
F4	413 ± 21	0.207 ± 0.05	95.41 ± 1.06
F5	810 ± 118	0.320 ± 0.04	47.81 ± 2.16
F6	645 ± 81	0.137 ± 0.17	64.87 ± 1.09
F7	452 ± 24	0.44 ± 0.08	98.60 ± 2.23
F8	1701 ± 97	0.373 ± 0.071	52.24 ± 1.33
F9	746 ± 67	0.325 ± 0.12	95.18 ± 2.35
F10	396 ± 93	0.421 ± 0.01	96.21 ± 3.21
F11	345 ± 12	0.240 ± 0.045	99.74 ± 5.06
F12	1651 ± 95	0.654 ± 0.235	55.31 ± 1.20
F13	1800 ± 105	0.954 ± 0.120	62.2 ± 3.54

* Niosome vesicle size after hydration of proniosomes. Values are expressed as mean ± (SD).

**Table 4:** Steady state transdermal flux “SSTF”, Enhancement ratio “ER” and permeability coefficient “PC” of curcumin permeated across rat skin.

Formula	SSTF ^a^ μg/cm^2^ × h	PC ^b^ cm^2^/h	Enhancement Ratio (ER) ^c^
**Curcumin suspension**	0.331 ± 0.087	0.00057 ± 0.0002	1
**F7**	7.072 ± 0.961 *	0.0122 ± 0.0016	21.86 ± 2.85
**F9**	8.46 ± 1.31 *	0.0146 ± 0.002	27.54 ± 11.52

^a^ SSTF = amount of permeated curcumin/time × area of permeation “4.91 cm^2^”. ^b^ PC = amount of permeated curcumin/time × saturation solubility of curcumin in sink medium “2850 µg/mL”. ^c^ (ER) = SSTF of formulation/SSTF of control (curcumin suspension). Values are expressed as mean ± (SD). * *p* < 0.05 compared to suspension form.

**Table 5:** Curcumin permeation kinetics from suspension form and niosomal formulations.

Formula	Zero Order		First Order		Higauchi Model		Korsmeyer-Peppas	
	R^2^	K_0_	R^2^	K_1_	R^2^	K_H_	R^2^	*n*
**Curcumin suspension**	0.6537	1.240	0.2707	0.0765	0.8999	7.2453	0.9786	0.2945
**F7**	0.9827	33.577	0.3959	0.1631	0.9538	171.86	0.9916	0.5568
**F9**	0.9754	38.468	0.3112	0.1490	0.970	199.3	0.9821	0.7345
